# Development, Design, and Electrical Performance Simulation of Novel Through-Type 3D Semi Spherical Electrode Detector Based on SOI Substrate

**DOI:** 10.3390/mi16091006

**Published:** 2025-08-31

**Authors:** Zhiyu Liu, Tao Long, Zheng Li, Xuran Zhu, Jun Zhao, Xinqing Li, Manwen Liu, Meishan Wang

**Affiliations:** 1 School of Integrated Circuits, Ludong University, Yantai 264025, China; 17861822572@163.com (Z.L.); longtao@ldu.edu.cn (T.L.); 18663488310@163.com (X.Z.); zhaojun@ldu.edu.cn (J.Z.); mswang1971@163.com (M.W.); 2 Engineering Research Center of Photodetector Special Chip in Universities of Shandong, Ludong University, Yantai 264025, China; xqli1996zz@163.com; 3 School of Materials Science and Engineering, Xiangtan University, Xiangtan 411105, China; 4 Institute of Microelectronics, Chinese Academy of Sciences, Beijing 100029, China; liumanwen@ime.ac.cn

**Keywords:** silicon detector, trench electrode, electrical characteristics, full depletion voltage, SOI

## Abstract

This article proposes a novel three-dimensional trench electrode detector, named the through-type three-dimensional quasi-hemispherical electrode detector. The detector adopts a trench structure to package each independent unit and achieves complete penetration of trench electrodes with the help of an SOI substrate. The horizontal distances from the center anode of the detector to the trench cathode and the detector thickness are equal. It has a near-spherical structure and exhibits spherical-like electrical performance. In this study, we modeled the device physics of the new structure and conducted a systematic three-dimensional simulation of its electrical characteristics, including the electric field, electric potential, electron concentration distribution of the detector, the inducted current caused by incident ions, and the crosstalk between detector units. Computational and technology computer-aided design (TCAD) simulation results show that the detector has an ultra-small capacitance (2.7 fF), low depletion voltage (1.4 V), and uniform electric field distribution. The trench electrodes electrically isolate the pixel units from each other so that the coherence effect between the units is small and can be applied in high-resolution X-ray photon counting detectors to enhance the contrast-to-noise ratio of low-dose imaging and the detection rate of tiny structures, among other things.

## 1. Introduction

Silicon detectors are important detectors widely used in fields such as nuclear physics [1], high-energy physics [2], and medical imaging [3]. In the field of nuclear physics, silicon detectors are widely used in particle detection and physical experiments, such as particle identification [4], particle trajectory reconstruction [5], metal element analysis [6], etc. In the field of high-energy physics, silicon detectors are widely used in large-scale collision experiments [7,8] such as CMS [9] and ATLAS [10] for the study of particles such as the Higgs boson [11]. In the medical field, silicon detectors are also widely used in medical imaging technologies such as positron emission tomography (PET) for tumor diagnosis, cardiac lesion detection, etc. [12,13]. Traditional silicon detectors are fabricated using planar technology (two-dimensional detectors), with electrodes being made on the surface of the wafer, resulting in longitudinal depletion. Compared to two-dimensional silicon detectors, the electrodes of three-dimensional detectors are directly fabricated in silicon materials, and the depletion thickness of the detector no longer depends on the wafer thickness. The depletion voltage can be controlled by designing the electrode spacing. Compared to two-dimensional detectors, three-dimensional detectors have superior radiation resistance, more optimized edge design, and more optimized charge collection efficiency. At present, the particle density of the High-Luminosity Large Hadron Collider (HL-LHC) has significantly increased, which has increased the spatial resolution requirements of the detector and promoted the miniaturization of pixel size [14]. At the same time, reducing the electrode spacing becomes key, which can shorten the charge drift path, alleviate charge trapping caused by radiation-induced defects, and enhance radiation hardness. The thickness of detectors tends to be thinner, which can reduce capacitance and adapt to micro electrodes, laying the foundation for performance optimization.

In 1997, Parker et al. from the University of Hawaii proposed the first generation of three-dimensional column electrode silicon detectors [15]. Compared to 2D detectors, the electrode spacing is not limited by the thickness of the chip, resulting in lower full depletion voltage, a shorter charge collection time, better radiation tolerance, and controllable electrode spacing, which improves mechanical stability and radiation resistance [16,17]. However, the detector suffers from a saddle-shaped potential (“dead zone”) in the center region of the electrodes and an uneven distribution of the electric field, which is concentrated in the vicinity of the collector electrodes [18]. In 2006, an important milestone was achieved with the invention of “active-edge” sensor technology by Kenney et al. [19]. Their pioneering work solved the key problem of dead zones around the physical boundaries of silicon sensors. Kenney’s technology utilizes deep reactive ion etching (DRIE) to create smooth, vertically defined edges. By doping these etched-out trenches and passivating them with a thermal oxide layer, they successfully transformed the mechanical edges into functional sensitive electrodes. This allows the sensitive volume to extend to within a few micrometers of the physical boundary, a significant improvement that is critical for applications where sensors need to be spliced with minimal dead space. However, despite the excellent resolution of the sensor perimeter by the active edge technique, the internal electrode structure and the associated bulk electric field distribution are still based on planar geometries or columnar electrodes in some 3D designs and thus, it is still structurally a planar 2D detector or 3D column electrode detector. These internal structures may still lead to inhomogeneous electric fields, low-field-strength regions, and limitations in further reducing the depletion voltage and readout capacitance [19]. In 2009, Brookhaven National Laboratory (BNL) proposed an innovative three-dimensional trench electrode silicon detector structure [18] which uses a vertical trench structure and a composition of central columnar electrodes. By optimizing the charge collection path, the signal charge collection rate and charge gain performance were significantly improved. Theoretical analysis and experimental results show that the three-dimensional groove electrode structure can achieve uniform electric field distribution inside the detector and effectively reduce the fully depleted operating voltage [20]. However, in order to prevent detachment of the detector cell, some unetched areas will be retained at the bottom, which leads to less utilization of the space inside the detector. In the following years, a breakthrough in the field of edge processing technology for silicon detectors was achieved by the VTT Technical Research Center of Finland. They developed an edge activation process based on ion implantation (instead of the conventional polysilicon filling technique) to prepare edge-free detectors with dead layer thickness below 1 μm on 6-inch wafers [21,22]. The technique achieves edge electrical activation by sidewall ion implantation, which avoids complex steps, such as polysilicon growth and planarization, and significantly improves fabrication efficiency and yield [21,23].

In the recent research on 3D trench detectors, most research institutes have made a lot of effort in the related process and structural design. For example, Forcolin et al. prepared a TIMEPIX-compatible 3D trench electrode pixel sensor for the first time in the framework of the INFN TIMESPOT project using a single-sided process at FBK, and they improved the problem of the uneven electric field of traditional 3D detectors by optimizing the geometry of the trench, which showed that its leakage current was as low as 10 pA/pixel and its capacitance was 70–75 fF/pixel. The optimization of trench geometry improves the problem of uneven electric fields in traditional 3D detectors, and the test shows that the leakage current is as low as 10 pA/pixel, the capacitance of the counter electrode is 70–75 fF/pixel, and the matching test beam experimentally achieves a timing resolution of less than 30 ps. At the same time, it points out that the 13% dead volume of the trench structure and the complexity of the process are the challenges of mass production [24]. In order to deal with the fabrication yields caused by the defects of long trench lithography, Ye et al. proposed an improved 3D trench design by introducing a 10 µm gap in the p^+^ ohmic trench and arranging it in a staggered manner with the n^+^ readout trench. TCAD simulations and Monte Carlo simulations show that this design has a minimal effect on the uniformity of the electric field and the weighting field, even when the trench is irradiated at 2 × 10^16^ neq/cm^2^, which is the same as that in the p^+^ ohmic trench [25]. Liu et al. focused on the irradiation resistance of 3D trench detectors on a N substrate. TCAD simulations show that when the electrode spacing is controlled at 5–20 µm, the full depletion voltage of the detector remains below 500 V and the transient current is lower than 500 V even under extreme irradiation. The TCAD simulation shows that when the electrode spacing is controlled at 5–20 µm, even under extreme irradiation injection, the full depletion voltage of the detector is still lower than 500 V and the peak response time of transient current is <100 ps, which confirms that the small electrode spacing is key to improve the irradiation resistance and provides a basis for the design of applications in a high-radiation environment [26].

In another paper published by our team [27], a new type of detector was designed using a double-sided etching and filling process, which effectively prevents the detector unit from falling off, thus realizing a feed-through electrode design and expanding the internal effective sensitivity area, but the process steps in this way are relatively large. This paper combines this design with the SOI process, also realizes the design of penetrating electrodes, and the whole process uses a single-sided process, which significantly simplifies the process steps; however, the price of SOI wafers is relatively high.

## 2. Three-Dimensional Semi Spherical Electrode Detector Based on SOI Substrate

**Figure 1:**
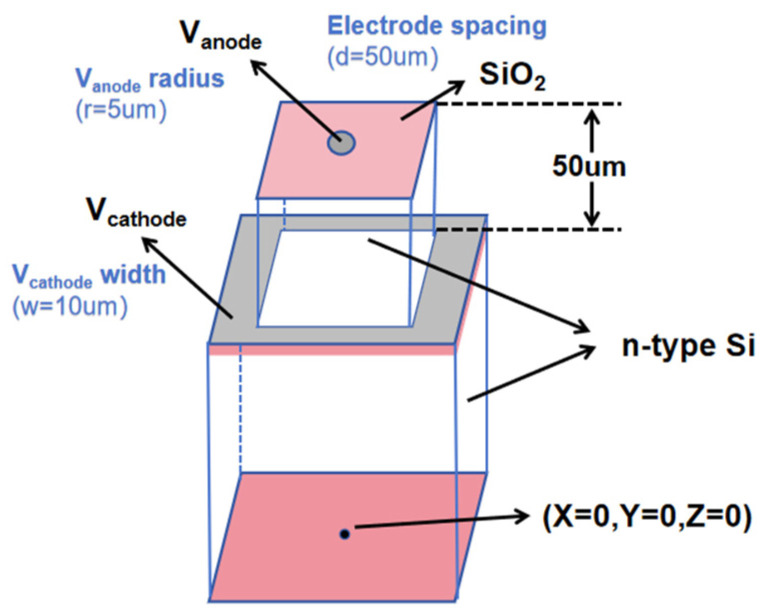
Conceptual diagram of detector.

Building on the inspiration from the aforementioned detectors, we propose a 3D hemispherical electrode detector based on an SOI substrate, which further innovates upon the 3D electrode structure, representing a true 3D silicon detector. This design, combined with an SOI (silicon-on-insulator) substrate, achieves a quasi-hemispherical electrode design with five sides of cathodes (four sides of doped trench electrodes and one bottom side with an ion implant electrode) surrounding a point-like collection anode for each pixel cell (as shown in Figure 1). It is entirely different from the detector structure presented in [19], where trench walls are present only in the peripheral regions of the detector chip to form an active edge. Our 3D hemispherical electrode detector array is a full 3D detector, whereas [19] uses an active slot wall to surround the entire 2D strip detector. Our new detector offers a more symmetrical electric field distribution than that of the 3D cylindrical trench electrode [20] and 3D column electrode detectors [15]. The small pixel design, where trench electrodes surround a central point-like anode, enables the new detector to have a smaller depletion voltage, smaller readout capacitance, and anti-crosstalk characteristics. The detector unit’s cell structure comprises a central point-contact collector electrode and a hemispherical electrode, wherein the hemispherical electrode consists of a peripheral groove electrode (as shown in Figure 1) and an ion-implanted back electrode. The detector achieves 2D position sensitivity through the layout of the array of this unit cell (3D pixel detector). Since cell capacitance is positively correlated with the area and depth of the collection electrode, the point-like collection electrode in our detector can significantly reduce the capacitance of each individual pixel (capacitance modeling, calculations, and simulation validation results will be provided later). Furthermore, such an enclosed design isolates each pixel from others, thus effectively eliminating charge sharing between detector cells. Therefore, this semi-spherical electrode detector has the advantages of low depletion voltage, uniform electric field distribution, small capacitance, and no charge-sharing effect between the detector pixel units, making it suitable for application in high-energy physics (radiation hard) and X-ray detection, such as particle colliders, security check, nuclear safety, and other scenarios. It may meet the requirements for future upgrades and replacements in ATLAS and CMS in the High-Luminosity (HL) Large Hadron Collider.

**Figure 2:**
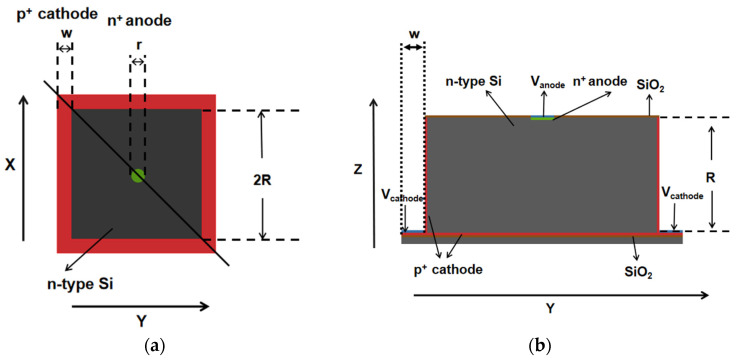
(**a**) Top view; (**b**) sectional view.

The detector unit has a rectangular structure, and its silicon substrate is lightly doped n-type with a doping concentration of 1 × 10^12^/cm^3^. Figure 2 shows the schematic diagram of the detector structure, and Figure 2a shows that the pixel units are separated by groove electrodes with a groove width of w. The dot-shaped central anode is arranged at the top of the detector with a radius of r. The vertical distance from the center area of the detector to the groove wall is R. Figure 2b shows that the trench is etched to the P-type heavily doped layer of the SOI substrate, with a trench depth of R. The trench cathode forms the P-type heavily doped layer through an ion implantation process. The cathode of the detector is composed of a trench wall and a P-type heavily doped layer on the substrate, resulting in a distance of R from the center anode of the detector to the trench cathode and the bottom cathode, forming a detector with a structural radius of R. Both the anode and cathode have a thickness of 1 µm and are covered with 1 µm aluminum electrodes on top.

Through theoretical calculations, we derive and compute the full depletion voltage and capacitance values of the novel detector. The reliability will be subsequently verified by simulation experiments.

The depletion voltage formula for a hemispherical electrode detector is [28]

(1) $$V_{fd}=\frac{eN_{eff}d^{2}}{6\varepsilon_{r}\varepsilon_{0}}+V_{bi}$$

where q is the charge of each electron, $e=1.6\times 10^{-19}\;C;\varepsilon_{r}$ is the relative dielectric constant of silicon, $\varepsilon_{r}=11.9;\;\varepsilon_{0}$ is the vacuum dielectric constant,${\;\varepsilon }_{0}=8.854\times 10^{-12}\;F/m$; *d* is the electrode spacing, d=50 μm; $N_{eff}$ is the effective doping concentration of N-type light doping on the silicon substrate,${\;N}_{eff}=1\times 10^{12}/{cm}^{2};$ and $V_{bi}$ is the built-in electric potential of the Si unit,${\;V}_{bi}=0.75\;V$. The depletion voltage of the detector can be obtained by using Equation (1), which is 1.4 V.

For hemispherical structures, according to Gauss’s theorem:

(2) $$E\left(r\right)A\left(r\right)=\frac{Q}{\varepsilon_{r}\varepsilon_{0}}$$

where $E\left(r\right)$ is the electric field at radius *r* and the surface area of the hemisphere at radius *r* is

(3) $$A=2\pi r^{2}$$

Therefore, we have

(4) $$E\left(r\right)=\frac{Q}{2\pi \varepsilon_{r}\varepsilon_{0}r^{2}}$$

Therefore, the electric potential difference from the cathode to the anode can be calculated as

(5) $$∆U=\int_{r_{0}}^{R}\frac{Q}{2\pi \varepsilon_{r}\varepsilon_{0}}\frac{dR}{r^{2}}=\frac{Q}{2\pi \varepsilon_{r}\varepsilon_{0}}\left(\frac{1}{r_{0}}-\frac{1}{R}\right)$$

The capacitance of the hemispherical detector is

(6) $$C=\frac{Q}{∆U}=\frac{2\pi \varepsilon_{r}\varepsilon_{0}r_{0}\;R}{R-r_{0}}$$

where *R* is the electrode spacing, R=d=50 μm, and $r_{0}$ is the center anode radius, $r_{0}=5\;\mu m$. By substituting the size of the detector into Equation (6), the capacitance of the detector can be approximately solved as 3.7 fF.

## 3. Electrical Characteristics

### 3.1. Analysis of Electric Field Characteristics

**Figure 3:**
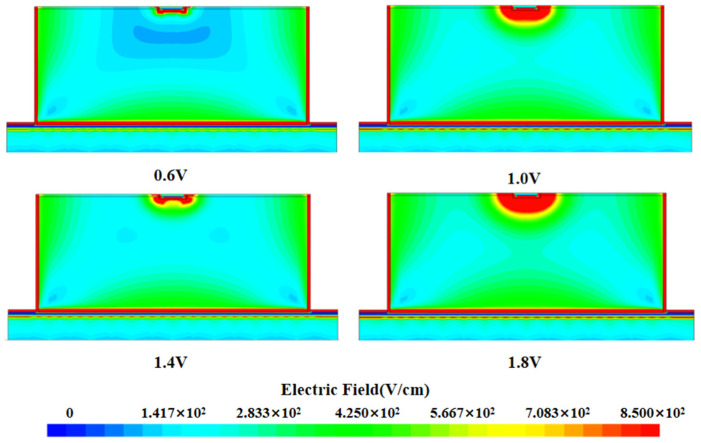
Two-dimensional electric field distribution at X = 0 for three-dimensional quasi-hemispherical electrode detectors at different voltages.

In the field of silicon detector research, the electric field distribution characteristics are one of the key factors in evaluating its performance. By analyzing the two-dimensional cut images obtained under different bias voltage conditions (as shown in Figure 3), we systematically observed the dynamic behavior of the electric field distribution inside the detector with voltage changes. Specifically, the low-electric-field region inside the detector decreases with the gradual increase in the applied bias voltage, and when the bias voltage reaches a certain threshold, i.e., when the detector is completely depleted, the internal electric field covers the whole detector and the zero-electric-field region disappears. When the voltage is −1.4 V or so, the detector is completely depleted, and at this time, there is almost no low-electric field or zero-electric-field region inside the detector. This also means that the carriers can always maintain a directional motion under the action of the electric field force, which enhances the collection efficiency of the carriers and thus improves the response speed and sensitivity of the detector.

**Figure 4:**
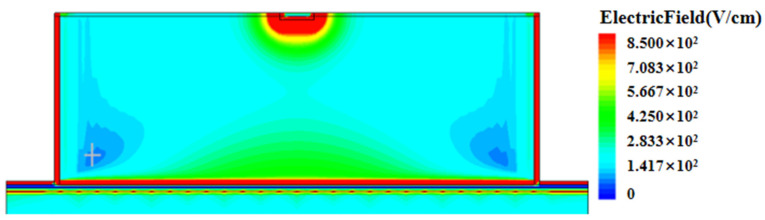
Electric field cross-section at the corner of the detector under 1.4 V voltage (the cut surface is at the diagonal in Figure 2a).

**Figure 5:**
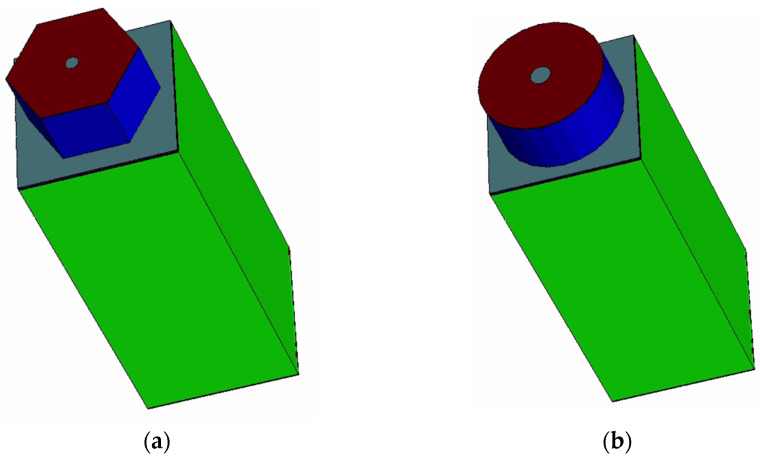
Other unit structures of the detector. (**a**) Hexagonal prism structure; (**b**) cylindrical structure.

Figure 4 shows a cross-section view of the electric field distribution in the corner region of the detector biased at a voltage of 1.4 V. It can be seen from the figure that the corner region is in a depleted state with a non-zero electric field. Although its electric field strength (at about 100 V/cm) is lower compared to that of the surrounding region, it still has the ability to make carriers to drift for charge collection. In applications where fast charge collection is needed, one can improve the low-electric-field situation by using a hexagonal cell shape for a detector array or a circular one for a single detector, as shown in Figure 5.

### 3.2. Analysis of Electron Concentration Inside Detector

**Figure 6:**
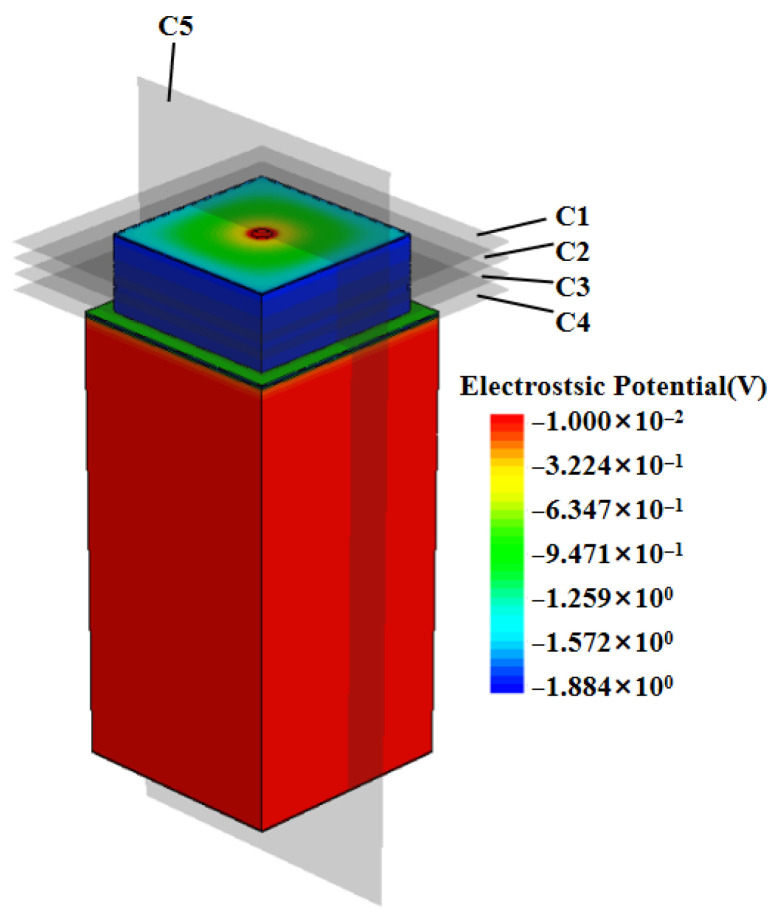
Detector unit potential diagram.

As shown in Figure 6, this study conducted a systematic simulation analysis of the electric potential distribution characteristics of a three-dimensional quasi-hemispherical detector.

**Figure 7:**
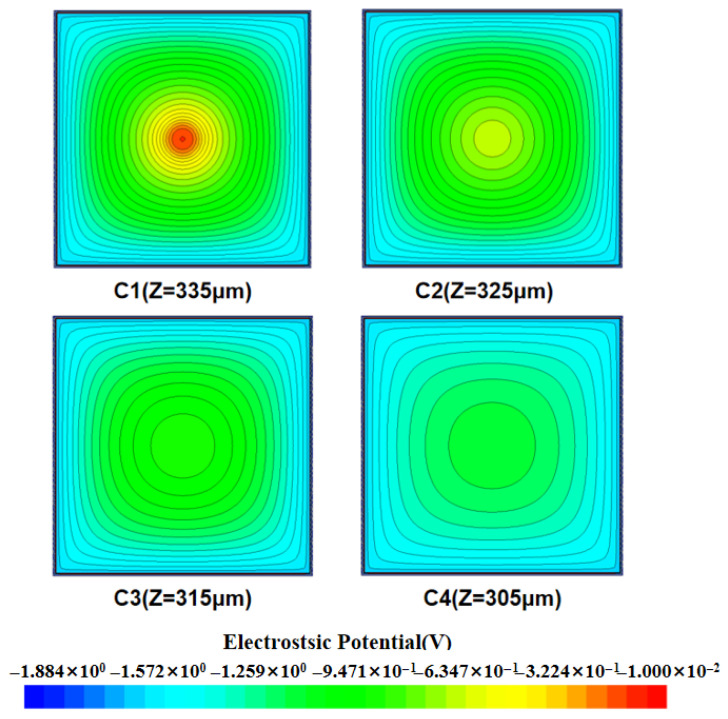
Two-dimensional potential distribution map at different heights of the detector.

Figure 7 presents the transverse cross-section of the detector’s electric potential distribution, revealing a significant ring-shaped characteristic in its internal electric potential (the simulated height is 340 um, the detector substrate height is 290 um, and the detector unit height is 50 um). The potential gradient is smooth and relatively uniform, and the potential shows a concentric-circle symmetric distribution, which means that the detector’s electric field in the symmetric direction as well as the motion of the carriers are consistent, improving the overall stability and reliability of the detector and also proving that the collection of incident particles by the detector is not affected by the angle.

### 3.3. Analysis of Electron Concentration Characteristics

**Figure 8:**
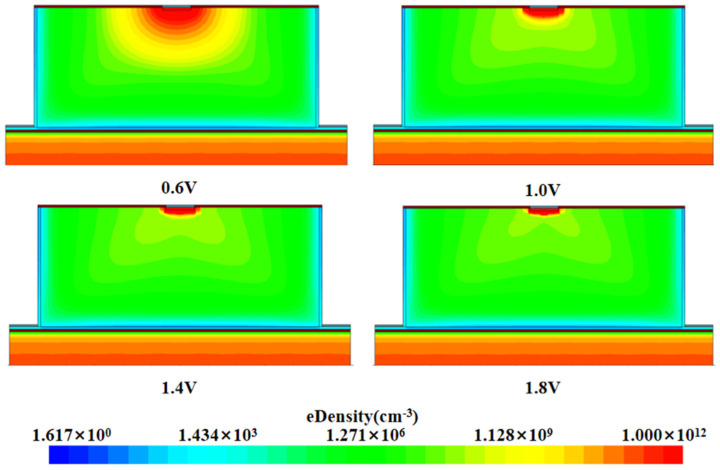
Two-dimensional electron concentration distribution at X = 0 for 3D quasi-hemispherical detectors at different voltages.

Figure 8 illustrates the distribution of electron concentration inside the detector at different bias voltages. It can be observed from the figure that the electron concentration near the cathode region is relatively low, while the electron concentration near the center collector region is significantly high. As the bias voltage increases, the electron concentration inside the detector decreases gradually. When the bias voltage reaches 1.4 V, the electron concentration inside the detector is already one to two orders of magnitude below its intrinsic doping concentration. And as the bias voltage continues to increase, the electron concentration inside the detector basically no longer changes, a phenomenon that indicates that the detector has reached a state of complete depletion.

**Figure 9:**
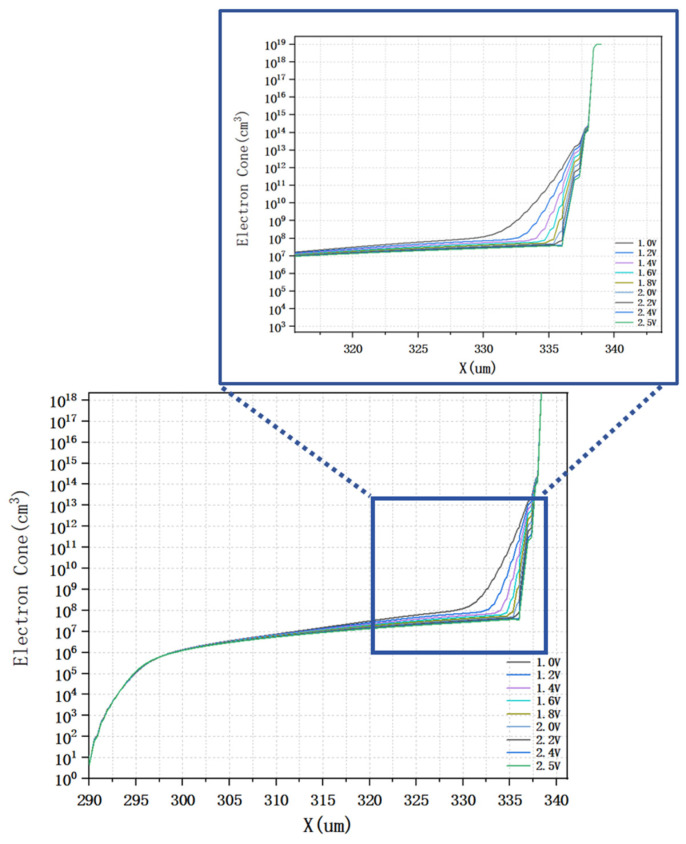
Electron concentration distribution at different voltages.

As illustrated in Figure 9, the one-dimensional electron density distribution curve is presented as a function of the *Z*-axis direction. The results demonstrate that, with an increase in the applied voltage, there is a consistent decrease in the electron density within the detector, until it reaches a specific threshold voltage. At this threshold voltage, the electron density within the detector approaches a state of complete exhaustion. When the applied voltage is equal to −1.4 V, the electron concentration in most of the effective sensitive region inside the detector is one to two orders of magnitude lower than the substrate concentration. As the voltage is increased further, the electronic concentration within the effective detection area remains constant, indicating that the detector has reached a state of complete depletion. Consequently, the depletion voltage of the detector is determined to be 1.4 V, which is consistent with the previous theoretical calculation results.

### 3.4. Analysis of Transient Induced Current in Detectors and Estimation of Charge Collection Efficiency After Irradiation

**Figure 10:**
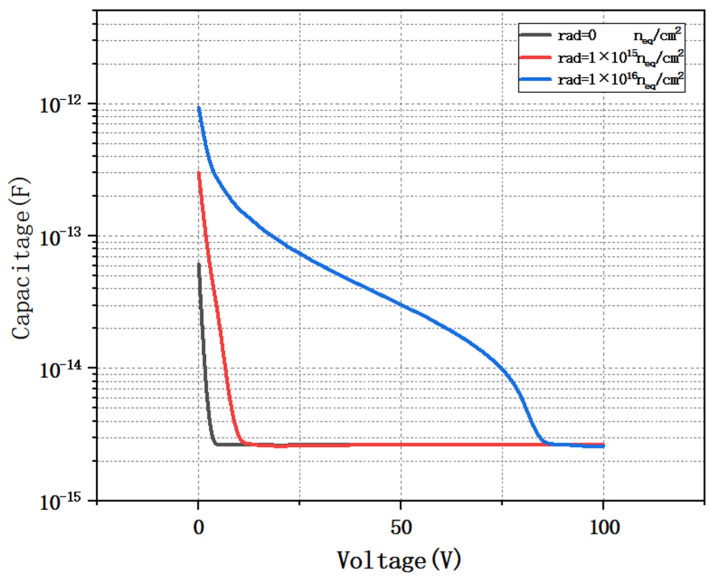
Capacitance curves of the detector at different irradiation injections.

As shown in Figure 10, we simulate the capacitance curves of the detector under different irradiation injections by varying the substrate doping concentration of the detector.

When the detector is irradiated by neutrons and charged particles, the effective doping concentration $N_{eff}$ will increase linearly with the 1 MeV neutron-equivalent flux $\varphi_{neq}$ at high fluxes as (N-type silicon)

(7) $$N_{eff}≅N_{d0}e^{-\gamma \varphi_{eq}}-\beta \varphi_{eq}$$

where $N_{d0}$ is the initial doping concentration, γ is the donor impurity removal rate, β is the deep acceptor introduction rate, and $\varphi_{eq}$ is the 1 MeV neutron equivalent flux. Since the *γ* of proton radiation is about 1 × 10^−13^ cm^2^ and β here is 0.01 /cm [29], in the case of $\varphi_{eq}$ > 1 × 10^14^ $neq/{cm}^{2}$,

(8) $$N_{eff}≅-\beta \varphi_{eq}$$

**Figure 11:**
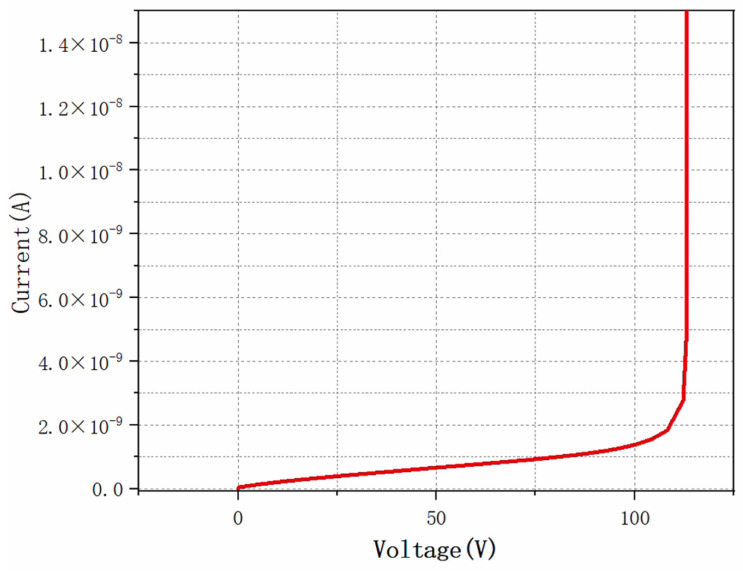
Detector breakdown characteristic diagram.

It can be clearly seen from the figure that when the detector reaches the fully depleted state, its capacitance value tends to stabilize and no longer varies with the increase in bias voltage. The capacitance characteristic of the detector is one of the key factors affecting its noise performance. Therefore, the smaller the capacitance, the less the noise. From the simulation results, it can be seen that the depletion voltage of the detector increases as the radiant flux increases. This is because the increase in radiative flux strongly alters the effective doping of the detector substrate [30]. Since the geometrical capacitance only depends on the physical structure of the detector, the saturation capacitance value usually does not change even if the depletion voltage of the detector changes when it is affected by external factors such as radiation. The simulation results further show that the depletion voltage of the detector remains around 88 V when the irradiation flux reaches 1 × 10^16^ cm^−2^, which fully verifies the feasibility of the detector to work under a strong irradiation environment. Figure 11 shows the breakdown characteristics of the detector. The breakdown voltage of the detector is 113 V.

### 3.5. Analysis of Transient Induced Current in Detectors

**Figure 12:**
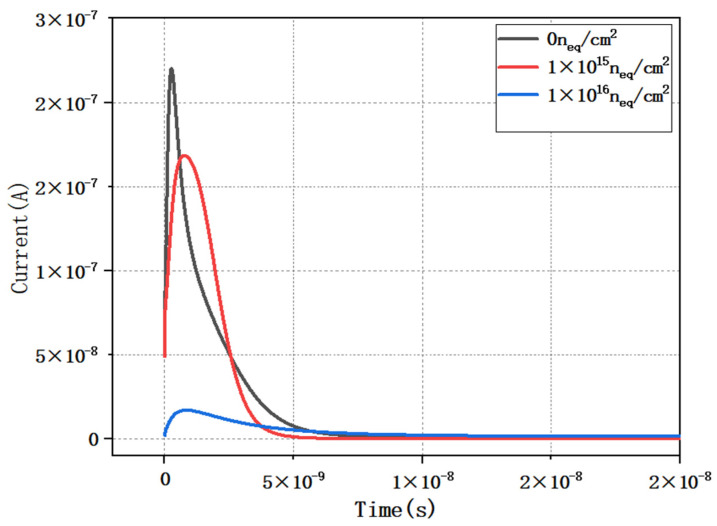
Instantaneous response current under different irradiation conditions.

As shown in Figure 12, we simulated the induced current profile of the detector under different irradiation injections by varying the substrate doping concentration of the detector. It can be clearly seen that the induced current of the detector peaks at around 1.6 ns under unirradiated conditions. It is also noted that the peak value of the induced current tends to decrease with increasing irradiation fluence. This phenomenon can be attributed to the damaging effect of irradiation on the detector material. As a result of irradiation, more defects are created inside the detector, which become trapping centers for charge carriers, thus reducing the charge collection efficiency. Below, we can simply estimate the collection time of incident particles for the detector at an operating voltage of 10 V based on the equation.

The average internal electric field of the detector is

(9) $$E=\frac{V}{d}$$

Therefore, the average drift response time of carriers in the detector is

(10) $$t=\frac{d}{\mu E}$$

In TCAD space, the following models were activated in the simulated incident particle collection simulation experiment: DopingDependence, eHighFieldSaturation, hHighFieldSaturation, Enormal, and CarrierCarrierScattering. The ion incident point was set to (0, 40, 339). The heavy-ion injection pulse duration was 1 × 10^−12^ s, with an implantation depth of 40 μm and a linear energy transfer (LET) of 1.28 × 10^−5^ pC/μm^2^. The operating voltage was set to -10 V, and the oxide charge density was converted to 4 × 10^11^ cm^−2^.

μ is the electron mobility, $\mu =1450\;{cm}^{2}/\left(V\cdot S\right)$. By substituting the detector size into Eq(10), the induction time is approximately 1.6 ns, which is roughly equivalent to the simulation results in Figure 12.

**Figure 13:**
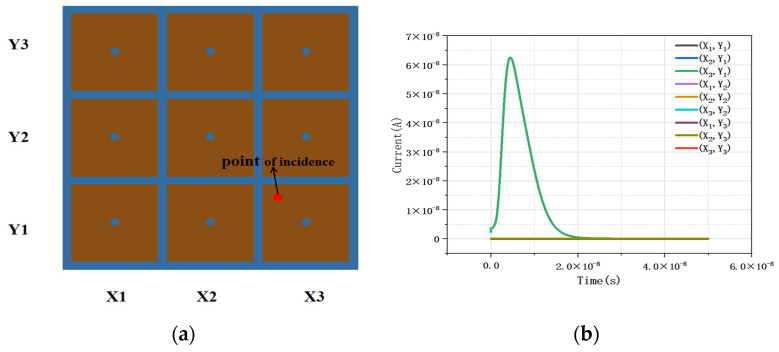
Instantaneous response current of incident particles in a 3 × 3 array (**a**) at the incident position and (**b**) response curve.

Figure 13 shows the instantaneous induced current curve of a 3 × 3 detector array. We simulated MIP (minimum ionizing particle) incidence on the lower right unit of the detector $\left(X_{3},Y_{1}\right)$ and obtained the induced current. Through observation, we found that no induced current occurred in adjacent units, only in the middle unit with MIP incidents, indicating that there is little crosstalk between detector units.

## 4. Conclusions

In this study, we present for the first time the structure of a novel detector, named the novel through-type 3D semi-spherical electrode detector based on an SOI substrate. This detector has the advantages of low depletion voltage, ultra-small capacitance, and symmetric distribution of a uniform electric field. In addition, its hemispherical structure ensures that the charge collection is almost independent of the angle θ, thus effectively capturing X-ray signals. It is an ideal X-ray detection tool, capable of effectively detecting hard X-rays and gamma rays, and it thus meets the requirements of high sensitivity and fast response in scenarios such as security check and nuclear safety. It also meets the requirements for future upgrades and replacements in the High-Luminosity (HL) Large Hadron Collider at ATLAS and CMS.

We simulated both individual cells and arrays of detector cells using Senturous TCAD 2019. We obtained the electrical characteristics of the detector such as potential, electric field, electron concentration distribution, capacitance, and incident particle induced current. The following conclusions were drawn:

The new detector utilizes a silicon-on-insulator (SOI) substrate and introduces a feedthrough electrode structure. This design significantly increases the effective sensitivity region of the detector. The full depletion voltage of the detector is measured to be 1.4 V, its geometrical capacitance is about 2.7 fF under non-irradiated conditions, and the response time of the detector is 1.6 ns at a bias voltage of 10 V with an electrode spacing of 50 μm. Simulation analysis shows that the detector array cells are effectively electrically isolated from each other, and the crosstalk can be neglected to ensure the high-energy-resolution performance of the device.

## Data Availability

The original contributions presented in the study are included in the article, further inquiries can be directed to the corresponding author.
